# Supplementary material for: COZOID: contact zone identifier for visual analysis of protein-protein interactions
Source: BMC Bioinformatics. 2018 Apr 6;19:125. doi: 10.1186/s12859-018-2113-6 (PMC5889581; doi:10.1186/s12859-018-2113-6)
Supplement: Supplementary file 2 — Software build. Executable binary file of the software tool. (ZIP 118,784 kb) [file 12859_2018_2113_MOESM2_ESM.zip › cozoid/java/docs/beansbinding-1.2.1-doc.zip!/org/jdesktop/beansbinding/class-use/PropertyStateEvent.html]

Uses of Class org.jdesktop.beansbinding.PropertyStateEvent (Beans Binding)


---


|  |  |  |
| --- | --- | --- |
| **Overview** \| **Package** \| **Class** \| **Use** \| **Tree** \| **Deprecated** \| **Index** \| **Help** | |  |
| PREV   NEXT | **FRAMES**    **NO FRAMES**     **All Classes** |  |


---


## **Uses of Class org.jdesktop.beansbinding.PropertyStateEvent**

| Packages that use PropertyStateEvent | |
| --- | --- |
| **org.jdesktop.beansbinding** | Provides support for defining properties and creating bindings between sets of two properties. |

Uses of PropertyStateEvent in org.jdesktop.beansbinding

| Methods in org.jdesktop.beansbinding with parameters of type PropertyStateEvent | |
| --- | --- |
| `protected  void` | `PropertyHelper.firePropertyStateChange(PropertyStateEvent pse)`             Notify listeners that the state of this property has changed, as characterized by the given `PropertyStateEvent`. |
| `void` | `PropertyStateListener.propertyStateChanged(PropertyStateEvent pse)`             Called to notify the listener that a change of state has occurred to one of the `Property` instances upon which the listener is registered. |
| `void` | `BindingListener.sourceChanged(Binding binding, PropertyStateEvent event)`             Notification that the source property of a `Binding` has fired a `PropertyStateEvent` for the `Binding's` source object. |
| `void` | `AbstractBindingListener.sourceChanged(Binding binding, PropertyStateEvent event)`             Notification that the source property of a `Binding` has fired a `PropertyStateEvent` for the `Binding's` source object. |
| `protected  void` | `Binding.sourceChangedImpl(PropertyStateEvent pse)`             Called to indicate that the source property has fired a `PropertyStateEvent` to indicate that its state has changed for the source object. |
| `protected  void` | `AutoBinding.sourceChangedImpl(PropertyStateEvent pse)` |
| `void` | `BindingListener.targetChanged(Binding binding, PropertyStateEvent event)`             Notification that the target property of a `Binding` has fired a `PropertyStateEvent` for the `Binding's` target object. |
| `void` | `AbstractBindingListener.targetChanged(Binding binding, PropertyStateEvent event)`             Notification that the target property of a `Binding` has fired a `PropertyStateEvent` for the `Binding's` target object. |
| `protected  void` | `Binding.targetChangedImpl(PropertyStateEvent pse)`             Called to indicate that the target property has fired a `PropertyStateEvent` to indicate that its state has changed for the target object. |
| `protected  void` | `AutoBinding.targetChangedImpl(PropertyStateEvent pse)` |

---


|  |  |  |
| --- | --- | --- |
| **Overview** \| **Package** \| **Class** \| **Use** \| **Tree** \| **Deprecated** \| **Index** \| **Help** | |  |
| PREV   NEXT | **FRAMES**    **NO FRAMES**     **All Classes** |  |


---
